# Hypervirulent *Klebsiella pneumoniae* causing aortitis retains its capsule and mucoviscosity and remains genotypically and phenotypically stable over time

**DOI:** 10.1038/s41598-025-23563-1

**Published:** 2025-11-13

**Authors:** Thomas A. Russo, Ulrike Carlino-MacDonald, Zachary J. Drayer, Connor J. Davies, Alan Hutson, Ting L. Luo, Melissa J. Martin, Patrick T. McGann, Francois Lebreton, Alan Sanders

**Affiliations:** 1 The Veterans Administration Western New York Healthcare System, 3495 Bailey Ave, Buffalo, NY 14215 USA; 2 Department of Medicine, Buffalo, USA; 3 Department of Microbiology and Immunology, Buffalo, NY USA; 4 The Witebsky Center for Microbial Pathogenesis, University at Buffalo, State University of New York, Buffalo, NY USA; 5 Department of Biostatistics and Bioinformatics, Roswell Park Comprehensive Cancer Center, Buffalo, NY USA; 6 Multidrug-Resistant Organism Repository and Surveillance Network (MRSN), Walter Reed Army Institute of Research, Silver Spring, MD USA; 7 Department of Medicine, Division of Infectious Diseases, Albany Medical Center, Albany, NY USA; 8 Jacobs School of Medicine and Biomedical Sciences, 955 Main St, Room 5212, Buffalo, NY 14203 USA

**Keywords:** Infection, Pathogens

## Abstract

Aortitis due to hypervirulent *Klebsiella pneumoniae* (hvKp) in a Belarusian male from central New York is described. Isolates at the time of diagnosis (Kp031824-1, Kp031824-2) and after 12 weeks of antimicrobial therapy (Kp070124) were characterized. Kp070124 was genomically and phenotypically unchanged retaining its capsular polysaccharide and mucoviscosity. In vitro studies established that capsule minus derivatives of Kp031824-1 and Kp031824-2 can occur due to mutations in *wcaJ*. But this genotype/phenotype was not selected for in Kp070124 after ≥15 weeks in the systemic compartment. Compared to capsule minus derivatives, the capsule positive phenotype demonstrated resistance to phagocytosis, but not to complement mediated bactericidal activity, suggesting resistance to phagocytosis is a more important defense mechanism at this site of infection. These data also support that a capsule positive, mucoviscous phenotype is selected for during infections in the systemic compartment. The surprising result that capsule positive strains have increased sensitivity to complement mediated bactericidal activity compared to capsule minus strains requires further investigation. The duration of therapy for this syndrome remains unclear but should be prolonged; adjunctive therapies (e.g. phage therapy, passive immunization, augmentation of cell mediated bactericidal activity) may be needed to overcome the protection endowed by the mucoviscous capsule of hvKp.

**Supplementary Information:**

The online version contains supplementary material available at 10.1038/s41598-025-23563-1.

## Introduction

 Hypervirulent *Klebsiella pneumoniae* (hvKp) is an emerging pathogen of increasing concern. The trademarks of hvKp infections are its ability to cause infections in the ambulatory setting with hepatic abscess and pneumonia being most common, multiple sites of infection, and unusual sites of infection compared to classical *K. pneumoniae* such as endophthalmitis, central nervous system infections, and necrotizing fasciitis^1^.

Although hvKp has been described to cause infection in nearly every site of the body, reports on vascular infection/mycotic aneurysm have been rare with cases primarily described from the Asian Pacific Rim^2–4^. Further, in established and putative cases due to hvKp^5–7^, genomic and phenotypic assessment of the offending strain, when performed, was limited to some combination of a string test, the identification of sequence type, capsule type, and 1–2 biomarkers. Given the rarity of this infection, optimal management, including the duration of antimicrobial therapy is uncertain, and in one case relapse occurred despite 5 weeks of antimicrobial therapy^4^.

In addition, there are a few reports in which serial hvKp isolates have been obtained from patients over time and assessed^8,9^. These data, combined with data from classical *K. pneumoniae* (cKp) have promoted the hypothesis that in the systemic compartment there is a positive selection pressure for the presence of capsule or increased capsule production, whereas in the urinary tract, selection pressure favors the loss of capsule^10^. However, data is limited and inconsistent^8,11,12^.

This report expands upon the existing knowledge base in three areas. First, we describe a case of aortitis due to hvKp for which the offending isolates underwent extensive genomic and phenotypic characterization. Second, a serial isolate from the aorta was recovered after 12 weeks of antimicrobial therapy and was also evaluated genomically and phenotypically. The aortic isolate was unchanged from the initial blood isolates despite a prolonged exposure to antimicrobial therapy; this suggests that for this systemic site either biofitness, which includes the presence of capsule, was optimized for survival or minimal replication occurred. Lastly the hvKp isolate was not eradicated despite a total of 12 weeks of antimicrobial therapy consistent with concerns that hvKp infections may require a longer treatment course than cKp. Taken together these data contribute to our understanding of the biology of hvKp infection and inform on management strategies needed for optimal outcomes.

## Results

### Clinical presentation and management of the aortitis

**Fig. 1:**
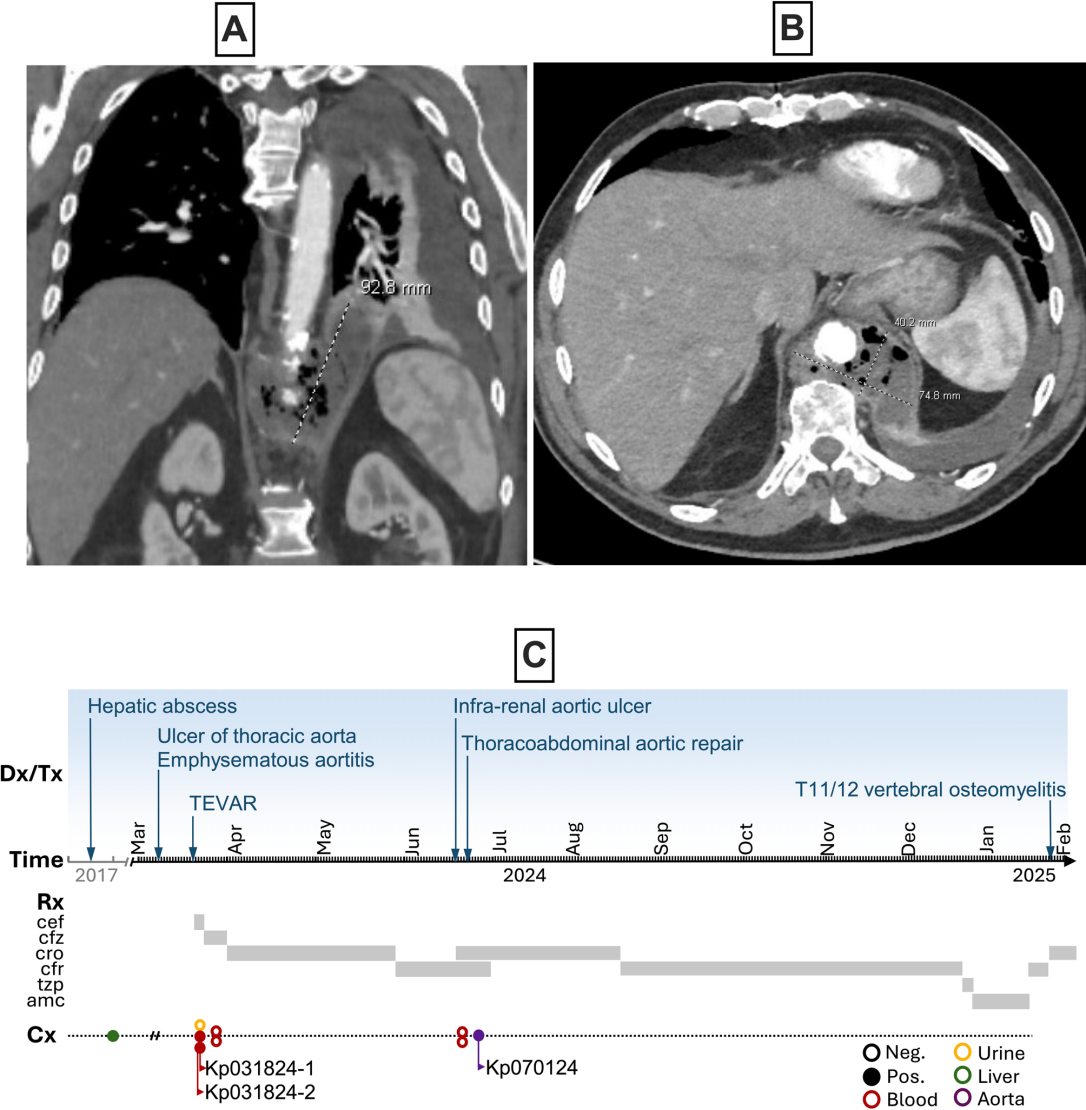
Clinical images of the aortitis and the timeline depicting infections, interventions, and strain isolation**.** *Panels a-b.* Computerized tomography images demonstrating emphysematous aortitis. Panel c. Timeline of the clinical course delineating the timing of the infectious syndromes, surgical interventions, antimicrobial treatments, and culture results. TEVAR = endovascular graft of the thoracic aorta, cef = cefepime, cfz = cefazolin, cro = ceftriaxone, cfr = cefadroxil, tzp = piperacillin-tazobactam, amc = amoxicillin-clavulanate.

A 73-year-old Belarusian male with a past medical history of diabetes, diverticulosis, hepatic abscess in 2017, chronic leukopenia (2–3 × 10^9^/L), benign prostate hyperplasia, colonic polyps, coronary artery disease s/p myocardial infarction and CABG, and hypertension presented to Wynn Hospital (Utica, New York) in March 2024 with complaints of fatigue, fever, epigastric abdominal pain, dysphagia, and occasional vomiting. He moved to central New York in 1989 and since has not travelled outside of the USA. A subdiaphragmatic ulcer of thoracic aorta and circumferential emphysematous thoracic aortitis was identified (Fig. 1A-B), which prompted transfer to Albany Medical Center (Albany New York) for further management. Upon admission, examination revealed a temperature of 102° F and vague abdominal pain without signs of peritonitis, and laboratory findings demonstrated a WBC of 4.86 × 10^9^/L, diabetic ketoacidosis, and a creatinine of 5.15 mg/dL. A urine culture was unrevealing but *Klebsiella pneumoniae*, resistant only to ampicillin, was isolated from 2 independent blood cultures. Initial treatment with cefepime was de-escalated to cefazolin. No growth occurred with subsequent blood cultures. A CAT scan of the abdomen and pelvis confirmed aortitis but did not reveal any coincidental focus of inflammation or abscess.

An urgent endovascular graft of the thoracic aorta (TEVAR) was performed to stabilize the intimal ulcer and what appeared to be an emerging leak without aneurysmal defect. Ceftriaxone 2 g IV q 12 h was initiated for ongoing treatment of the emphysematous aortitis. A right axial femoral bypass was performed to offload the heart and maintain retrograde visceral organ perfusion in anticipation of an eventual open aortic resection and reconstructive surgery.

An 8-week course of ceftriaxone was completed as an outpatient followed by approximately a 4-week course of cefadroxil 500 mg PO BID. While still on cefadroxil, about three months after initial presentation, the patient returned to hospital with fever to 102° F and pain in the mid abdomen and back. C-reactive protein was 188 mg/L. Although a CAT scan of the abdomen performed two weeks earlier had demonstrated near resolution of the large emphysematous peri-aortic collection, repeat imaging demonstrated a new penetrating infra-renal aortic ulcer, and persistent inflammation of the aorta that contained the TEVAR graft. Blood cultures revealed no growth, and IV ceftriaxone 2 g q 12 h was restarted. Four days after admission, he underwent thoracoabdominal aortic repair with a dacron tube graft, removal of the TEVAR graft, and a second infra renal aortic tube graft in the new area of ulceration. Other than a short post operative complication of spinal ischemia that resolved following lumbar drain decompression, the patient had an uneventful recovery. Notably, intra-operative culture of aortic tissue was positive for *Klebsiella pneumoniae*, with an identical sensitivity profile, as the blood culture isolates three months earlier.

The patient was discharged on hospital day 20 to complete eight weeks of IV ceftriaxone 2 g q 12 h. At the completion of this therapy, he was deemed to be in an excellent state of health and was started on an indefinite course of oral cefadroxil 500 mg BID. Three months later, a follow up CRP was 5.4 mg/L.

The patient was readmitted to Albany Medical Center in late December 2024 with acute sigmoid diverticulitis, without abscess, and treated medically with piperacillin-tazobactam for five (5) days followed by oral amoxicillin-clavulanate for 4 weeks; cefadroxil, was restarted in January 2025 after his treatment course with amoxicillin-clavulanate was completed.

However, in late January 2025, while on suppressive cefadroxil therapy, the patient represented with severe mid-back pain, and MRI imaging revealed discitis and vertebral osteomyelitis at the T11/12 region, which was dorsal to the Dacron aortic graft placed six (6) months earlier. Interventional radiology needle biopsy of the vertebral body yielded *Klebsiella pneumoniae* (isolate not available for study) with identical sensitivity pattern as all previous isolates. The patient was discharged on a 12-week course of IV ceftriaxone 2 g daily. The patient underwent an uneventful sigmoid resection in March 2025. Pathology revealed active diverticulitis with adhesions, and tissue culture grew vancomycin-resistant *Enterococcus faecium*, with no *Klebsiella* isolated. The patient was discharged from hospital to complete his 12-week course of IV ceftriaxone for his vertebral infection, with planned return to lifelong suppressive or cefadroxil therapy.

Interestingly, review of his remote medical records revealed that the hepatic abscess that was drained seven years prior to his emphysematous aortitis, yielded a *Klebsiella pneumoniae* with an identical sensitivity pattern (isolate unavailable). At that time the patient had evidence for coincidental sigmoid diverticulitis, CT scans also revealed significant chronic sigmoid diverticulosis raising the concern that this was a source for his hepatic infection. The patient was referred to colo-rectal surgery for consideration of a sigmoid resection, but surgery was not performed (see Fig. 1C for timeline of clinical events).

### The initial *K. pneumoniae* blood isolates possessed the hypervirulent genotype and phenotype

The two 3/18/2024 blood isolates Kp031824-1 and Kp031824-2 underwent whole genomic sequencing and analysis. Both isolates were genetically identical (0 SNPs apart), were sequence type (ST) 23, and possessed all 5 of the biomarkers present on the hvKp virulence plasmid that most strongly predict the hypervirulent phenotype (*iucA*,* iroB*,* peg-344*, *rmpA*,* rmpA2*)^13^. Using the pLVPK sequence as a reference, the 3 isolates shared a large alignment fraction (> 94.9%; difference largely due to the lack of a single 5 kb region present in pLVPK that contained 9 CDS of mostly unknown function excepting one that was predicted as a putative TraD) and > 99.7% nucleotide identity, confirming the presence of the pLVPK-like plasmid (Supplementary Figure S1). The chromosomal KL1 capsule biosynthetic locus was identified as well as ICEKp10, which contained the biosynthetic genes for yersiniabactin and colibactin. A core genome MLST analysis revealed that these isolates were not closely genetically related (> 10 allelic differences) to ST23 isolates in the public domain (Pathogenwatch analysis platform). The closest isolates were distinct by 13 allelic variants. At that threshold, we identified a total of 352 isolates collected from over 30 countries (Supplementary Figure S2 and Supplementary Table S1). No inferences on transmission routes could be made from this analysis.

**Fig. 2:**
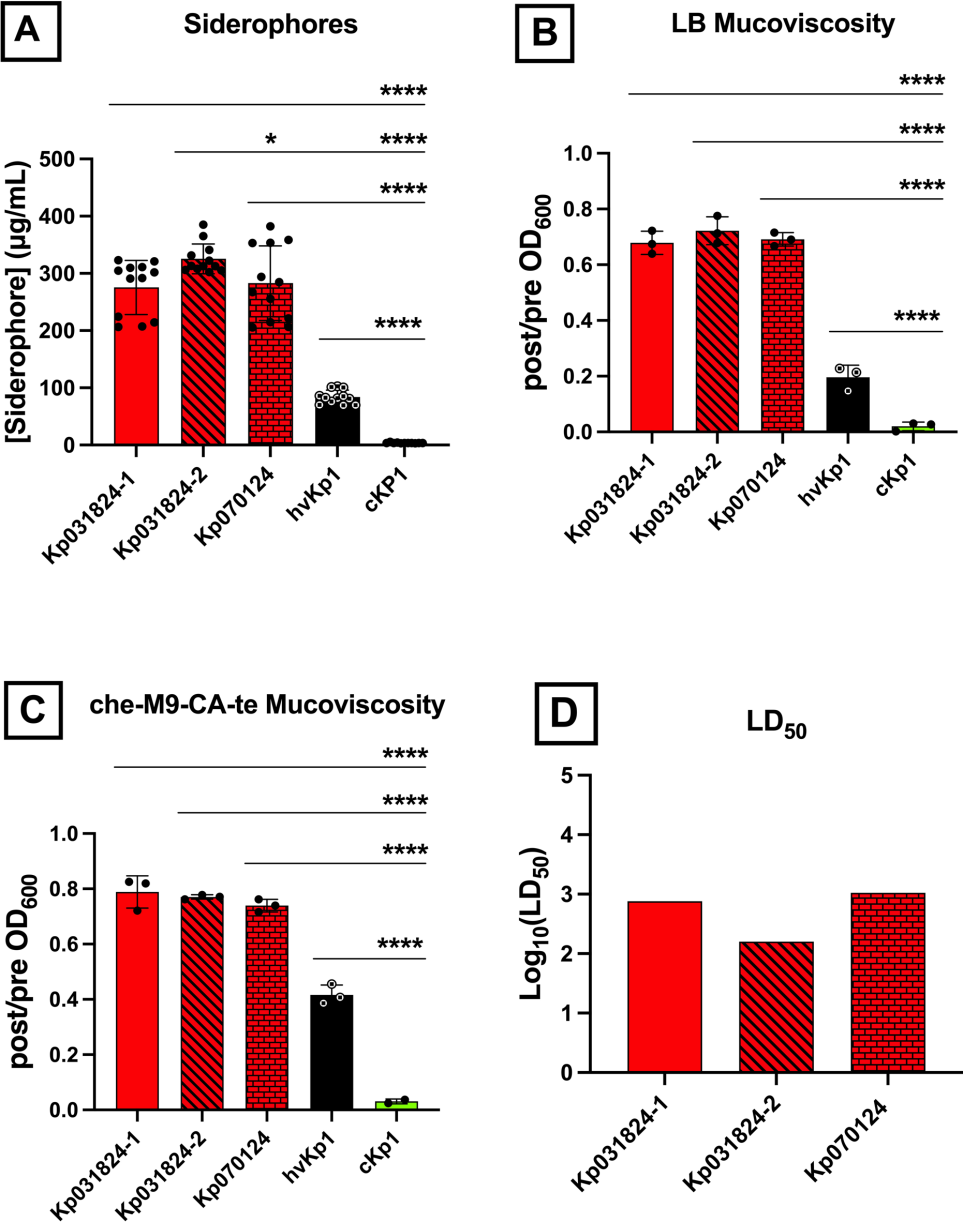
*In vitro* quantitative siderophore production, mucoviscosity, and *in vivo* virulence is similar between Kp031824-1, Kp031824-2, and Kp070124. *Panel a*. In vitro quantitative siderophore production for Kp031824-1, Kp031824-2, and Kp070124. Standards with concentrations of 0, 1.5, 3.1, 6.25, 12.5, 25, 50, and 100 µg/ml enabled quantitation. *Panels b-c*. In vitro quantitative mucoviscosity for Kp031824-1, Kp031824-2, and Kp070124 grown in lysogeny medium (5 g yeast extract, 10 g NaCl, 10 g tryptone, 15 g agar) (LB) and chelated minimal medium supplemented with casamino-acids and trace elements (che-m9-CA-te), respectively. A minimum of three biological replicates was performed for each strain for siderophore and mucoviscosity assays; hvKp1 and cKp1 were utilized as controls. Comparisons between Kp031824-1, Kp031824-2, and Kp070124 or hvKp1 and cKp1 were performed via ordinary one-way ANOVA, using Šidák’s multiple comparisons test (Prism 10.4.2 for MacIntosh, GraphPad Software Inc.). *Panel d*. Log_10_(LD_50_) values for Kp031824-1, Kp031824-2, and Kp070124. The LD_50_ was estimated using a logistic regression model with the factors for strain and inoculum (CFU/mL). A comparison between Kp031824-1, Kp031824-2, and Kp070124 was made by employing a blend of the empirical logit function along with least-squares regression incorporating strain and inoculum factors (CFU/mL) to derive p-values for comparing dose-response curves based on LS-means (SAS/STAT 15.1). All data except LD_50_ data are presented as the mean ± SD. ****p ≤ 0.0001, *p ≤ 0.05.

Next, quantitative siderophore production and mucoviscosity was assessed (Figs. 2a-c, Supplementary Tables S2, S3). Kp031824-1 and Kp031824-2 produced significantly higher levels of siderophores in M9 minimal medium containing casamino acids (c-M9-CA) (275.5 ± 47.28 µg/mL and 325.4 ± 26.22 µg/mL respectively) compared to the cKp control strain cKp1 (3.665 ± 0.650 µg/mL) (*p* < 0.0001). Likewise, Kp031824-1 and Kp031824-2 were significantly more mucoviscous in lysogeny medium (5 g yeast extract, 10 g NaCl, 10 g tryptone, 15 g agar) (LB) (post/pre OD_600_ 0.6788 ± 0.041 and 0.7221 ± 0.050 respectively) compared to the cKp control strain cKp1 (0.02 ± 0.016) (*p* < 0.0001) and in chelated minimal medium supplemented with casamino-acids and trace elements (che-m9-CA-te) (post/pre OD_600_ 0.789 ± 0.058 and 0.771 ± 0.008 respectively) compared to the cKp control strain cKp1 (0.031 ± 0.009) (*p* < 0.0001). Lastly, the LD_50_ for Kp031824-1 and Kp031824-2 for CD1 outbred mice that underwent SQ challenge were 7.63 × 10^2^ CFU and 1.59 × 10^2^ CFU respectively (Log_10_ LD_50_ values of 2.882 and 2.201 respectively); in this model hvKp strains have a Log_10_ value of < 7.0 (Fig. 2d). For reference the Log_10_ LD_50_ for hvKp1 is 2.879 and for cKp1 is > 8.0. Taken together genotypic and phenotypic data unequivocally demonstrate that Kp031824-1 and Kp031824-2 are hvKp isolates.

### The K. pneumoniae aortitis isolate after 12 weeks of antimicrobial therapy retained its capsular polysaccharide, mucoviscosity, and the hypervirulent phenotype

Despite 8 weeks of ceftriaxone and approximately 4 weeks of cefadroxil, intraoperative cultures obtained during thoracoabdominal aortic repair on July 1 st, 2024, resulted in the isolation of Kp070124. This isolate was present in this in vivo compartment for at least 106 days (the initial isolates were grown from blood obtained on March 18th, 2024). Genomic and phenotypic evaluation of this isolate would lend insight into in vivo selective pressures.

First, Kp070124 underwent whole genome sequencing and analysis, and these data were compared to the initial blood isolates Kp031824-1 and Kp031824-2. Surprisingly, no genomic changes were identified. Although this finding suggests that Kp070124, Kp031824-1, and Kp031824-2 should share an identical phenotype, transcriptional or post-transcriptional differences could not be excluded. Therefore, several phenotypic evaluations were performed.

**Fig. 3:**
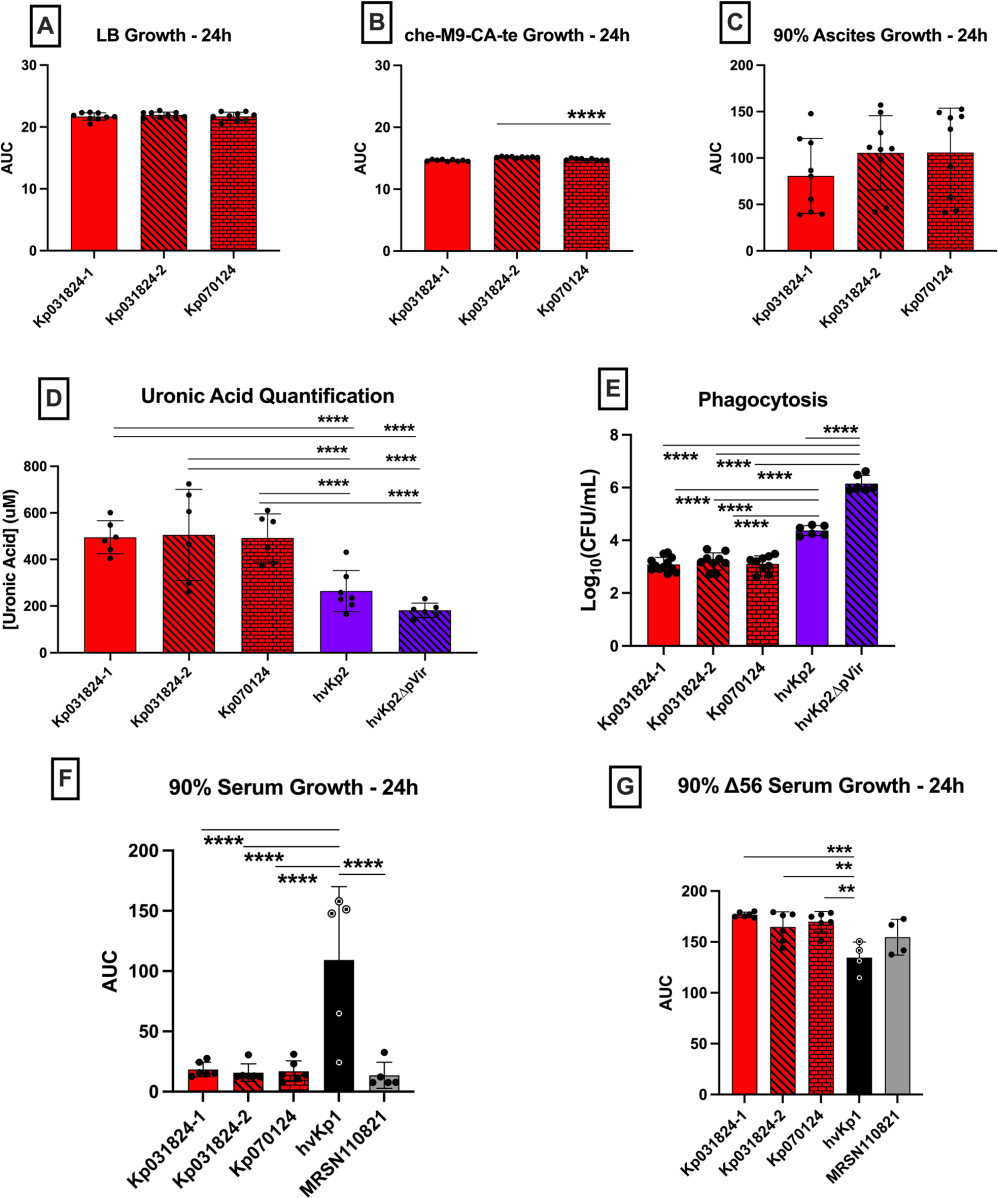
*In vitro* growth in LB, che-M9-CA-te, human ascites, human serum and complement inactivated serum, uronic acid production, and phagocytosis are similar between Kp031824-1, Kp031824-2, and Kp070124. *Panels a-c*. In vitro growth assessment of Kp031824-1, Kp0318234-2, and Kp070124 in LB, che-M9-CA-te and human ascites respectively. Growth in LB and che-M9-CA-te was measured by OD_600_ over 24 h and growth in ascites was measured by enumerating CFU, from which the area under the curve (AUC) was calculated. *Panel d.* In vitro quantitative uronic acid production. *Panel e.* In vitro quantitative assessment of phagocytosis by J774A.1 murine macrophages. Data presented as Log_10_(CFU/mL), which is derived from the difference in concentration of surviving bacteria in cytochalasin D-treated wells vs. untreated wells. *Panels f-g*. In vitro growth assessment in 90% human serum and ∆56 °C serum (complement-mediated bactericidal activity has been inactivated), respectively. Growth was measured via enumeration of colony-forming units (CFU) over 24 h, from which the AUC was calculated. A minimum of three biological replicates with three technical repeats was performed for each strain for all in vitro assays. hvKp1 and MRSN110821 (cKp strain) served as controls for serum assays, and hvKp2 and hvKp2∆pVir (same K1 capsule type as Kp031824-1, Kp031824-2, and Kp070124) were utilized as controls for uronic acid and phagocytosis assays. All data are presented as the mean ± SD. Comparisons between strains were made via ordinary one-way ANOVA, using Šidák’s multiple comparisons test (Prism 10.4.2 for MacIntosh, GraphPad Software Inc.). ****p ≤ 0.0001, ***p ≤ 0.001, **p ≤ 0.01.

First, growth and growth-related factors were assessed. The growth of Kp070124, Kp031824-1 and Kp031824-2 was similar in LB and numerically similar in c-M9-CA-te (Kp070124 versus Kp031824-2 was statistically but not biologically different) (Figs. 3a-b, Supplementary Figure S3, Supplementary Tables S2, S3). Further, Kp070124 produced similar levels of siderophores (283.1 ± 65.23 µg/mL) as Kp031824-1; Kp070124 and Kp031824-2 produced numerically similar levels that were statistically but not biologically different (Fig. 2a, Supplementary Tables S2, S3). Next, bacterial defense factors/properties were assessed. Kp070124 possessed similar mucoviscosity (post/pre OD_600_ 0.691 ± 0.024 and 0.740 ± 0.0230 respectively) as Kp031824-1 and Kp031824-2 in LB and c-M9-CA-te (Fig. 2b-c, Supplementary Tables S2, S3). Capsule production for these strains, which was measured by quantitating uronic acid, was also similar (Fig. 3d, Supplementary Tables S2, S3). Lastly, resistance to host-mediated bactericidal factors was determined. Kp070124 demonstrated a similar degree of resistance to both complement mediated bactericidal activity and macrophage mediated phagocytosis as Kp031824-1 and Kp031824-2 (Fig. 3e-g, Supplementary Figure S3, Supplementary Tables S2, S3). Lastly, an ex vivo and in vivo assessment was performed. The growth of Kp070124 was similar to Kp031824-1 and Kp031824-2 when grown ex vivo in human ascites (Fig. 3c, Supplementary Figure S3, Supplementary Tables S2, S3). Further, when CD1 outbred mice underwent SQ challenge with Kp070124, the LD_50_ (Log_10_ 3.021) was similar to that observed for Kp031824-1 and Kp031824-2, (Fig. 2d, Supplementary Tables S2-S4).

These data demonstrate that Kp070124 has retained its hypervirulent phenotype. Siderophore production, mucoviscosity, capsule production, resistance to complement mediated bactericidal activity and macrophage mediated phagocytosis, ex vivo growth in ascites, and lethality in a murine systemic infection model were similar compared to the initial isolates Kp031824-1, Kp031824-2 despite prolonged in vivo selection pressure over at least 106 days.

### Despite antimicrobial pressure for approximately 12 weeks no evolution in antimicrobial resistance was observed

It is not uncommon for antimicrobial resistance to evolve after a prolonged course of therapy. However, Kp070124 did not develop resistances nor were any genomic signatures predictive of resistance identified. The MIC for ceftriaxone against Kp031824-1, Kp031824-2, and Kp070124 as reported by the clinical microbiology laboratory was ≤ 1.0 µg/mL. To exclude small changes in resistance to ceftriaxone that may have conferred a selective advantage in the micro-environment of the infected aorta additional MIC testing of concentrations ranging from 0.00195 to 32 ug/mL was performed. However, no increase in the MIC for ceftriaxone against Kp070124 compared to Kp031824-1 and Kp031824-2 was observed (0.0625 µg/mL, 0.125 µg/mL, 0.125 µg/mL respectively).

### The initial *K. pneumoniae* isolates could evolve into capsule deficient derivatives

The observation that Kp070124 maintained a capsule supports that this phenotype is best suited for survival in this systemic compartment. However, this assumes that Kp031824-1 and Kp031824-2 could evolve a capsule minus phenotype. Therefore, Kp031824-1 and Kp031824-2 were assessed to determine the frequency of evolution to a capsule negative phenotype in vitro. Colonies of capsule positive bacteria appear yellow, whereas colonies of capsule negative bacteria appear grey. Therefore, 100–200 individual colonies of Kp031824-1 and Kp031824-2 were visually screened on LB agars plates after overnight growth in either LB or human ascites. After growth in LB, 2.1% and 3.1% of Kp031824-1 and Kp031824-2 colonies respectively appeared grey. After growth in ascites, 3.2% and 0% of Kp031824-1 and Kp031824-2 colonies respectively appeared grey. Conversely, grey derivatives appeared to be stable, reversions from a grey to yellow phenotype were not observed with serial passage.

**Fig. 4:**
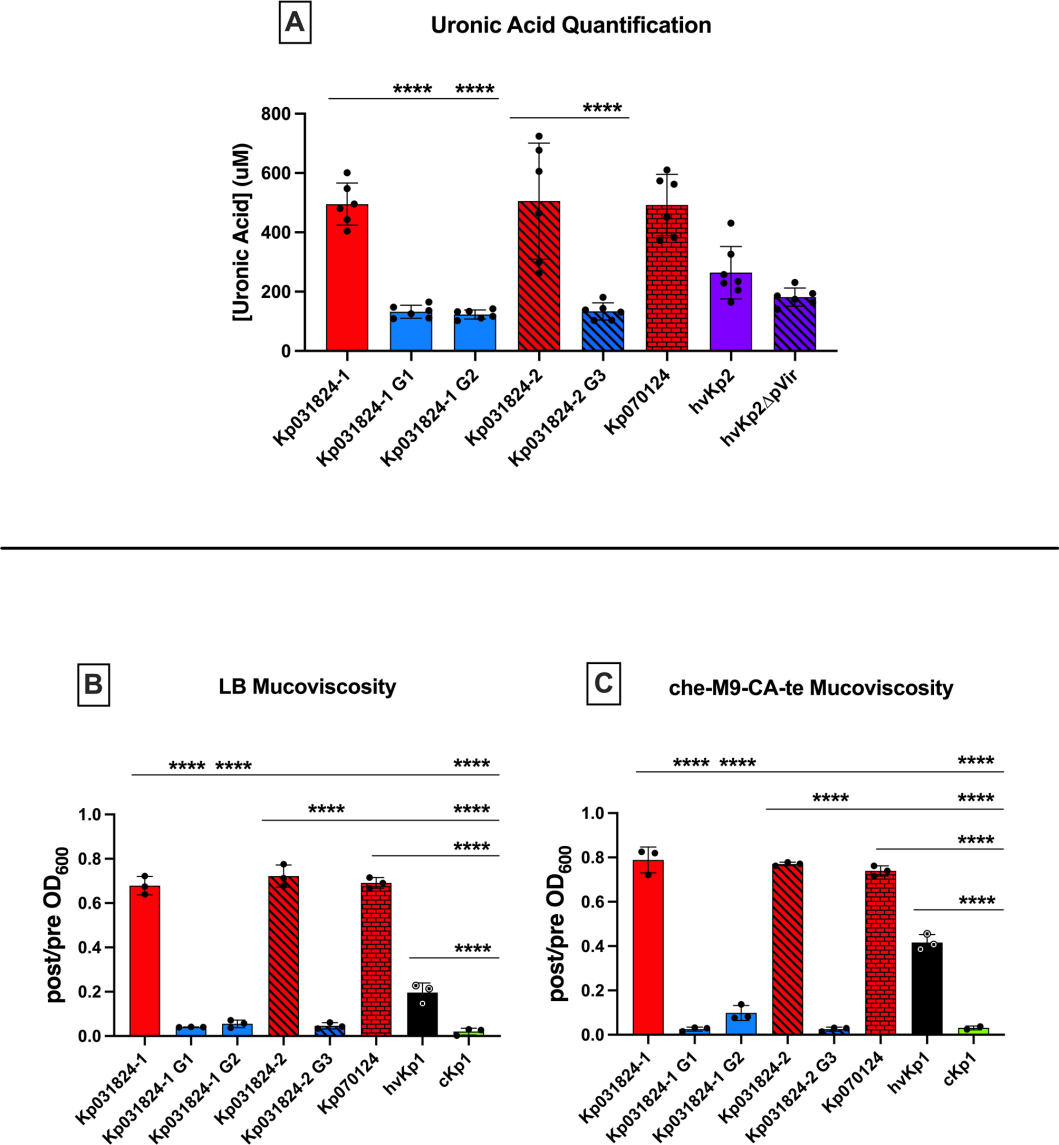
Kp031824-1, Kp031824-2, Kp070124 produce significantly more uronic acid and are significantly more mucoviscous than Kp031824-1 G1, Kp031824-1 G2, and Kp031824-2 G3. *Panel a.* In vitro uronic acid quantification for Kp031824-1, Kp031824-2, Kp070124, Kp031824-1 G1, Kp031824-1 G2, and Kp031824-2 G3. *Panel b-c.* In vitro quantitative mucoviscosity for Kp031824-1, Kp031824-2, Kp070124, Kp031824-1 G1, Kp031824-1 G2, and Kp031824-2 G3 grown in LB and che-m9-CA-te, respectively. A minimum of three biological replicates was performed for each strain. hvKp2 and hvKp2∆pVir were utilized as controls for the uronic acid assay, hvKp1 and cKp1served as controls for siderophore and mucoviscosity assays. All data are presented as the mean ± SD. Comparisons between strains were made via ordinary one-way ANOVA, using Šidák’s multiple comparisons test (Prism 10.4.2 for MacIntosh, GraphPad Software Inc.). ****p ≤ 0.0001.

Three grey derivatives of Kp031824-1 and Kp031824-2, Kp031824-1 G1 (obtained after growth in LB), Kp031824-1 G2 (obtained after growth in ascites), and Kp031824-2 G3 (obtained after growth in LB), were assessed to confirm that this colonial morphology reflected the loss of capsule. First, Kp031824-1 G1, Kp031824-1 G2, and Kp031824-2 G3 underwent whole genomic sequencing to determine the basis for the observed phenotype using their wild-type parents as the reference. All three capsule minus derivatives were isogenic with their wild-type parents except for a mutation in *wcaJ* which encodes the glycosyltransferase WcaJ, a critical protein requisite for capsule synthesis^14^; Kp031824-1 G1 had a stop codon at gln250, Kp031824-1 G2 had a val173glu substitution, and Kp031824-2 G3 had a Ile272 frameshift mutation leading to a premature stop codon at residue 287 (Supplementary Figure S4). To confirm that the identified mutations resulted in a significant decrease or loss of capsule, uronic acid was quantitated, which reflects capsule production. As expected, the mean uronic acid concentration was significantly less for Kp031824-1 G1, Kp031824-1 G2, and Kp031824-2 G3 (132.4 ± 21.82 µg/mL, 123.0 ± 15.43 µg/mL, 133.4 ± 28.85 µg/mL respectively) compared to their wild-type parents Kp031824-1 and Kp031824-2 (495.2 ± 70.95 µg/mL, 505.3 ± 195.5 µg/mL respectively, *p* < 0.0001) (Fig. 4a, Supplementary Tables S2, S3). Mucoviscosity is also dependent on capsule production^15^, and as expected, mucoviscosity was significantly decreased in Kp031824-1 G1, Kp031824-1 G2, and Kp031824-2 G3 compared to their wild-type parents after growth in both LB and c-M9-CA-te medium (*p* < 0.0001) (Fig. 4b-c, Supplementary Tables S2, S3). These values are similar to what was observed for the cKp control strain cKp1 (Figs. 4b-c, Supplementary Tables S2, S3). These data demonstrate that the grey colonial morphology reflects a significant decrease or loss of capsule and that Kp031824-1 and Kp031824-2 could evolve a capsule minus phenotype.

### Antimicrobial pressure does not appear to be a positive selection pressure for retention of capsule in vivo

If Kp070124 was more resistant to ceftriaxone than the capsule-minus derivatives Kp031824-1 G1, Kp031824-1 G2, and Kp031824-2 G3 this would suggest the antimicrobial pressure could select for a capsule-positive phenotype. However, this was not the case. The MIC for ceftriaxone against Kp070124 compared to Kp031824-1 G1, Kp031824-1 G2, and Kp031824-2 G3 was similar (0.0625 µg/mL, 0.0625 µg/mL, 0.125 µg/mL, 0.125 µg/mL respectively).

### Phagocytosis, but paradoxically not complement mediated bactericidal activity appears to be a positive selection pressure for retention of capsule in vivo

**Fig. 5:**
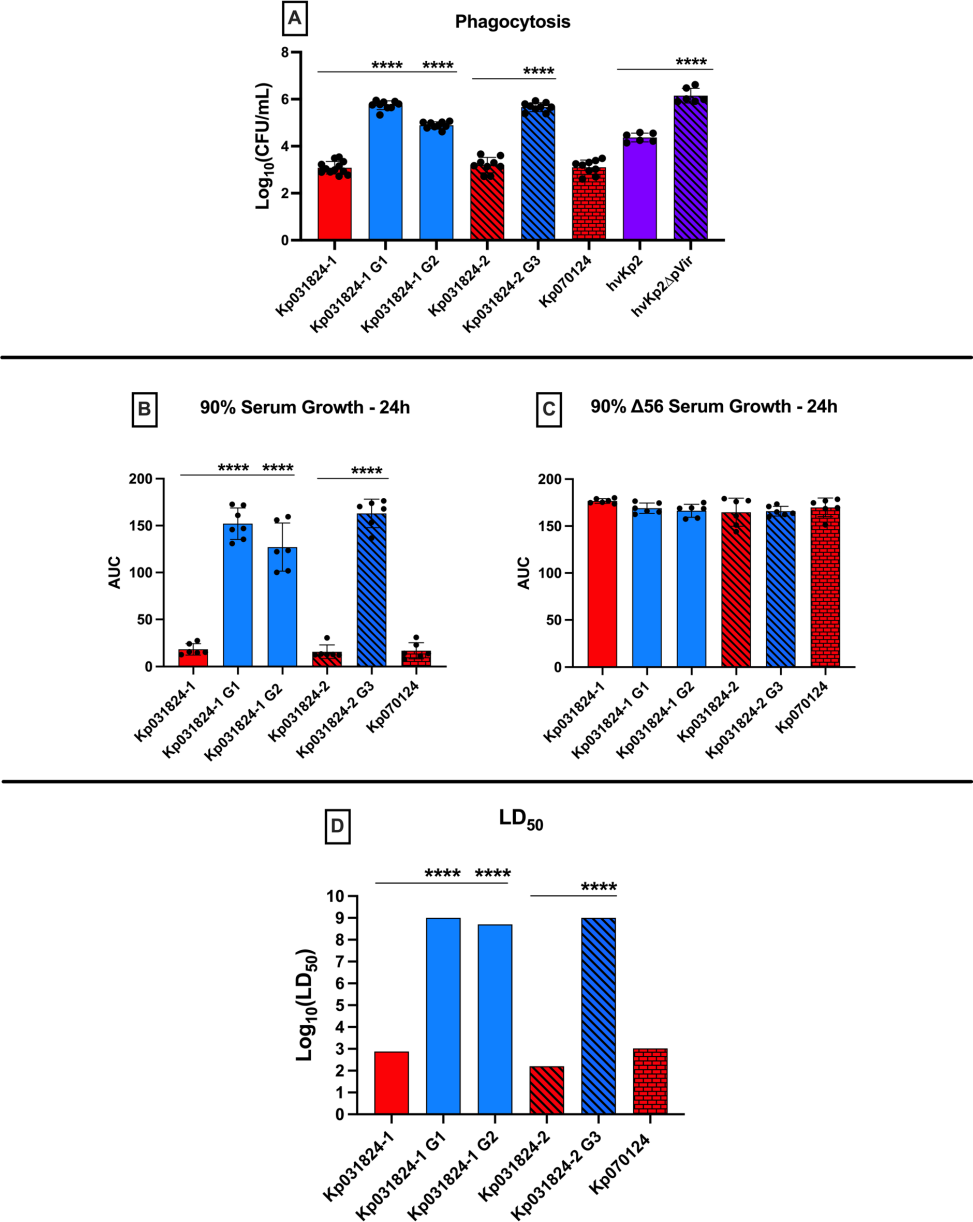
Kp031824-1, Kp031824-2, Kp070124 are more resistant to phagocytosis, more virulent *in vivo*, but less resistant to complement-mediated bactericidal activity than Kp031824-1 G1, Kp031824-1 G2, and Kp031824-2 G3. *Panel a.* In vitro quantitative assessment of Kp031824-1, Kp031824-2, Kp070124, Kp031824-1 G1, Kp031824-1 G2, and Kp031824-2 G3 phagocytosis by J774A.1 murine macrophages. Data presented as Log_10_(CFU/mL), which is derived from the difference in concentration of surviving bacteria in cytochalasin D-treated wells vs. untreated wells. *Panel b-c*. In vitro growth assessment of Kp031824-1, Kp031824-2, Kp070124, Kp031824-1 G1, Kp031824-1 G2, and Kp031824-2 G3 in 90% human serum and ∆56 °C serum, respectively. Growth was measured via enumeration of colony-forming units over 24 h, from which the AUC was calculated. *Panel d.* Log_10_(LD_50_) values for Kp031824-1, Kp031824-2, Kp070124, Kp031824-1 G1, Kp031824-1 G2, and Kp031824-2 G3. The LD_50_ was estimated using a logistic regression model with the factors for strain and inoculum (CFU/mL). Comparisons between strains were made by employing a blend of the empirical logit function along with least-squares regression incorporating strain and inoculum factors (CFU/mL) to derive p-values for comparing dose-response curves based on LS-means (SAS/STAT 15.1). An upper cutoff value of Log_10_(LD_50_) equal to 9 was employed to correct for biologically implausible LD_50_ values. A minimum of three biological replicates with three technical repeats was performed for each strain for all in vitro assays. hvKp2 and hvKp2∆pVir were utilized as controls for phagocytic uptake. All data excluding LD_50_ data are presented as the mean ± SD. Excluding LD_50_ data, all comparisons between strains were made via ordinary one-way ANOVA, using Šidák’s multiple comparisons test (Prism 10.4.2 for MacIntosh, GraphPad Software Inc.). ****p ≤ 0.0001, ***p ≤ 0.001, **p ≤ 0.01, *p ≤ 0.05.

Kp031824-1 and Kp031824-2 were compared to Kp031824-1 G1, Kp031824-1 G2, and Kp031824-2 G3 to generate mechanistic insights into which host defense factors mediated the positive selection for the retention of capsule within the systemic compartment. Not surprisingly the capsule positive wild-type parents Kp031824-1, Kp031824-2, and Kp070124 were significantly more resistant to phagocytosis than the capsule negative derivatives Kp031824-1 G1, Kp031824-1 G2, and Kp031824-2 G3 (Fig. 5a, *p* < 0.0001, Supplementary Tables S2, S3). By contrast, Kp031824-1, Kp031824-2, and Kp070124 were significantly more sensitive to complement mediated bactericidal activity than Kp031824-1 G1, Kp031824-1 G2, and Kp031824-2 G3 (Figs. 5b-c, *p* < 0.0001, Supplementary Tables S2, S3). Next, the LD_50_ for Kp031824-1 G1, Kp031824-1 G2, and Kp031824-2 G3 in CD1 outbred mice that underwent SQ challenge was measured. The LD_50_ for Kp031824-1 G1, Kp031824-1 G2, and Kp031824-2 G3 was > 7 (log_10_ CFU) (Fig. 5d, Supplementary Tables S2, S3), a dose commensurate with the cKp phenotype. These data support that within the systemic compartment, selection for resistance to phagocytosis, which is mediated by the presence of capsular polysaccharide, appears to be more important than increased resistance to complement mediated bactericidal activity, which is mediated by the loss of capsular polysaccharide.

## Discussion

Vascular infection/mycotic aneurysm due to *K. pneumoniae* is uncommon. *Staphylococcus aureus*, Streptococci, and Salmonella are the most common offending pathogens^16^. In this report, the most detailed genotypic and phenotypic assessment of a hvKp strain responsible for aortitis, including whole genomic sequence data, is described. It is unclear what proportion of *K. pneumoniae* mediated vascular infections/mycotic aneurysms is due to hvKp since most often the isolates have not been characterized. However, based on the clinical syndrome and/or strain characterization^2–4^ and an incomplete but suggestive evaluation of the isolate^5–7^ at least 4 cases due to hvKp and 3 cases putatively due to hvKp have been reported. From a clinical perspective, given the inability of the clinical microbiology laboratory to differentiate cKp from hvKp, it is best to assume that an hvKp isolate could be responsible for cases of vascular infection due to *K. pneumoniae* since this would affect management (e.g. vigilance for endophthalmitis, identifying occult abscesses)^13^.

Several additional clinical caveats can be gleaned from this report. First, hvKp infections have been most described in the Asian Pacific Rim and in individuals of Asian, Pacific Islander, and Hispanic ethnic background^1^. However, hvKp should not be excluded as the potential offending agent when these risk factors are absent as evidenced by this case. The infected Belarusian had resided in central New York since 1989 and had not travelled outside of the US. When and where this individual acquired the hvKp strain remains unclear, but there is some evidence that carriage of *K. pneumoniae* ST23/KL1 strains that possess all 5 of the biomarkers predictive of hvKp is occurring in healthly individuals^17^. However, until hvKp strains are routinely identified in the clinical microbiology laboratory, clinical suspicion and consideration is paramount. In addition, despite 12 weeks of antimicrobial therapy, hvKp was still isolated from the site of infection. There is uncertainty as to whether hvKp infections require a more prolonged course of treatment compared to cKp. No prospective trial data is available, but several reports have described relapses of non-vascular infection^18–21^, a mycotic aneurysm infection due to hvKp^4^, and a mycotic aneurysm due to an uncharacterized *K. pneumoniae* strain^22^. It is unclear if this is a unique characteristic of hvKp. However, biologically plausible mechanisms can be hypothesized that include the survival of the hvKp infecting strain at the site of initial infection due to its hypermucoviscous phenotype which could result in an in vivo biofilm even in the absence of foreign material^23,24^ or increased resistance to host factors such as professional phagocyte mediated bactericidal activity. Both scenarios could dictate the need for a more prolonged treatment course. In this case, one cannot exclude the possibility that failure was due to the presence of the endovascular graft. Lastly, it is intriguing that 7 years earlier the patient in this report had a liver abscess due to *K. pneumoniae*. That strain was unavailable for characterization, so it is unclear whether it was a hvKp isolate, and if so, whether it was the same as those in this report. However, if this isolate was a hvKp strain, it raises the question of whether hvKp has become part of the gastrointestinal flora as has been described^25–27^ and/or whether there is a genetic risk for infection.

Prior studies demonstrated that the loss or decreased production of capsule increases the ability to acquire exogenous DNA, including plasmids that assist in increased biofilm production, adherence to epithelial cells, and persistence in the murine urinary tract^9,28^. But by contrast, the loss or decreased production of capsule decreases resistance to macrophage-mediated phagocytosis and virulence in murine lethality infection models^9,12,28^. These data support the hypothesis that genotypic/phenotypic changes such as the loss of or decreased capsule production that favors adhesion and biofim formation enhance colonization and persistence in certain niches where the host innate defense factors are less active^29^. However, in systemic sites, such as the bloodstream, the presence of or increased capsule production protects against host-mediated bactericidal activity and increases survival. Since these data support that capsule producing strains produce less biofilm it appears that the ability to form a more robust biofilm is less important than resistance to phagocytosis in the systemic compartment. Data from this report are consistent with that hypothesis.

In this study, the capsule minus derivatives did not revert to the capsule positive phenotype after 6 passages, although this possibility cannot be excluded under different environmental conditions. Since the capsule positive phenotype is needed for extraintestinal survival^30,(and this study)]^ an inability of hvKp capsule minus strains to revert to a capsule positive phenotype would be problematic for hvKp to cause systemic infection. However recent data offers a potential explanation^31^. Chu et al. demonstrated that the transcriptional regulator IroP mediates changes in which under iron rich conditions biofilm formation and cell adhesion is increased whereas under iron poor conditions, such as the systemic compartment, mucoviscosity increases. The ability to transition between states that favor colonization and infection based on environmental cues is a potential explanation for this quandary.

The loss or decreased production of capsule in *K. pneumoniae* resulted in decreased complement-mediated bactericidal activity in some^11,28^, but not all studies^9^. Our finding demonstrated that capsule deficient derivatives due to mutations in *wcaJ* were more resistant to complement-mediated bactericidal activity. Wang et al. reported increased binding of the complement component C3b in similar capsule deficient derivatives^28^. However, how this translates into increased complement resistance is unclear. By contrast, the loss of capsule resulted in increased susceptibility to macrophage phagocytosis. Since in this study the capsule positive wild-type strain was selected for/retained in vivo in the human host and was needed for the hypervirulent phenotype in a mouse systemic infection model, these data support the concept that resistance to phagocytosis is a more critical determinant than resistance to complement mediated bactericidal activity for at least the endovascular systemic compartment. It would be important to determine if a capsule positive phenotype is also selected for at other extraintestinal sites because if so, then adjunctive therapeutic interventions directed against capsules can be designed (e.g. bacteriophage that use capsule as their receptor or passive immunization directed against capsular antigens).

It was somewhat surprising that despite 12 weeks of antimicrobial pressure, resistance did not develop. We compared the initial blood isolates Kp031824-1 and Kp031824-2 to the subsequent aortic tissue isolate Kp070124 for changes in the ceftriaxone MIC below the susceptibility breakpoint (< 1 µg/mL), but no change was observed. Possible explanations include some combination of poor penetration of cephalosporins at the site of infection, biofilm formation, which may have been facilitated by the endovascular graft, functional resistance due to bacterial persisters^32^, the development of small colony variants^33^, or intracellular survival^10^. But upon in vitro passage small colony variants were not observed. Further published data supports that at least in vitro, biofilm formation, cellular uptake and survival is facilitated by a capsule minus phenotype, contrasting the capsule positive of phenotype of Kp070124 ^8–10,12^. Therefore, it remains unclear why Kp070124 was able to persist within the human host despite ongoing antimicrobial treatment. However, if the increased capsule production and mucoviscosity inherent in hvKp strains is contributory, the use of rifampin as an adjunctive agent may be beneficial and warrants testing since it may decrease capsule production and mucoviscosity^34^.

The biggest limitation of this study is that this is a case report with only a single serial human isolate available for evaluation. Nonetheless, the findings contribute to our understanding of the biology of hvKp infection and inform on potential management strategies that require further assessment. Further, whenever clinical isolates are evaluated, there is a concern that subsequent passage may have inadvertently affected the genotype/phenotype. However, this seems unlikely to have occurred for Kp031824-1, Kp031824-2, and Kp070124 since whole genomic sequencing did not identify any genomic changes between isolates, therefore lending confidence that genomic fidelity was retained.

In summary, until clinical microbiology laboratories are capable of accurately identifying hvKp strains, the clinician needs to maintain a high degree of suspicion, regardless of the geographic location and ethnic background of the patient, to guide management^13^. Data that defines the risk of relapse/recurrence and the optimal duration of treatment for hvKp infections is needed. The determination of genotypic/phenotypic stability and the maintenance of capsule and mucoviscosity in hvKp serial isolates demonstrates the importance of this trait in the systemic compartment. Further, capsule promotes increased resistance to phagocytosis, but not complement mediated bactericidal activity compared to isogenic capsule minus derivatives. Taken together these data support that adjunctive therapies (e.g. phage therapy, antimicrobials, passive immunization, augmentation of cell mediated bactericidal activity) may be needed to overcome the protection endowed by mucoviscous capsular polysaccharide of hvKp.

## Methods

All methods were carried out in accordance with relevant guidelines and regulations. The procedures for obtaining human ascites fluid and serum were reviewed and approved by the Western New York Veterans Administration Institutional Review Board. Informed consent was obtained from all subjects donating blood for the isolation of serum. Informed consent for ascites fluid was waived because it was collected from de-identified patients who were undergoing therapeutic paracentesis for symptoms due to abdominal distension.

### Bacterial strains

Kp031824-1 and Kp031824-2 were independent *K. pneumoniae* blood isolates from the case patient. Kp070124 was a tissue isolate obtained during the resection of the aortic aneurysm. hvKp1, hvKp2, (hvKp strains), and cKp1, MRSN110821 (cKp strains) were variably used as controls for selected phenotypic assays and have been described^35^. hvKP2 and hvKp2ΔpVir (an isogenic derivative in which the hvKp virulence plasmid was cured, which results in decreased but not absent capsule production) were used and deemed more appropriate controls for the uronic acid and phagocytosis experiments because like Kp031824-1, Kp031824-2, and Kp070124 they produce a K1 capsule type whereas hvKp1 produces a K2 capsule type. Kp031824-1 G1 and Kp031824-2 G3 were spontaneous derivatives of Kp031824-1 and Kp031824-2 respectively that demonstrated a grey colonial morphology on agar plates after growth in lysogeny medium (5 g yeast extract, 10 g NaCl, 10 g tryptone, 15 g agar) (LB). This contrasts to the yellow colonial morphology of its progenitors Kp031824-1 and Kp031824-2. Kp031824-1 G2 was a spontaneous derivative of Kp031824-1 that demonstrated a grey colonial morphology on agar plates after growth in human ascites.

### Whole genome sequencing, de novo assembly, annotations, and variant analysis

All strains were sequenced using Illumina MiSeq short read platforms. Short read sequences were assembled *de novo* using Shovill (v1.0.9) with minimum assembly thresholds for contig size and coverage set at 200 bp and 49.5X, respectively. MLST assignment was performed using mlst v2.22.1 (https://github.com/tseemann/mlst). Comparisons of pLVPK plasmids were generated using the BLAST comparison tool version 1.4.1 integrated in Proksee.ca (PMID: 37140037). Parameters included: an expect value cutoff of 0.0001, filtering of low complexity regions, and filtering of regions with < 90% nucleotide identity. Single nucleotide polymorphism (SNP) calling was performed with Snippy v.4.4.5 (https://github.com/tseemann/snippy) using error corrected [Pilon v1.23] and annotated [Prokka v1.14.6] draft assemblies with isolate Kp031824-1 used as a reference. The core alignment length of the comparator isolates to the reference was 5.628 Mb. Snippy default parameters were used except for the minimum number of reads covering a site to be considered (--mincov − 20); and the minimum proportion of the reads which must differ from the reference (--minfrac − 80).

### Quantitative siderophore assay

Strains were grown overnight at 37 °C in iron-chelated M9 minimal medium containing casamino acids (c-M9-CA)^36^ and culture supernatants were assessed using the chromeazurol S dye assay as described^37^. Standards with concentrations of 0, 1.5, 3.1, 6.25, 12.5, 25, 50, and 100 µg/ml enabled quantitation. A minimum of 3 biological assays with 3 technical repeats were performed and the results were reported as the mean ± the SD.

### Quantitative mucoviscosity assay

Strains grown overnight in LB were used to inoculate 10 mL of either LB or c-M9-CA plus added trace elements (5 µg/mL CaCl_2_, 1 µg/mL CoCl_2_, 20 µg/mL MgCl_2_, 10 µg/mL MnCl_2_) (c-M9-CA-te) to a starting OD_600_ of approximately 0.2. Strains were grown at 37 °C for 24 h, and the assay was performed as described^38^. A minimum of 3 biological assays were performed and the results were reported as the mean ± the SD.

### Growth assessment

Growth assays were performed in either LB, c-M9-CA-te, ascites, or serum as described^39,40^. To obtain serum, blood obtained from healthy volunteers underwent coagulation at room temperature for 15 min, followed by refrigeration at 4 °C for 60 min to enable clot retraction. Serum was obtained after subsequent centrifugation at 4 °C for 15 min at 3,000 × *g*. A portion of this serum was heated at 56 °C for 30 min to inactivate complement mediated bactericidal activity (∆56 °C serum). For LB growth experiments, bacteria from an overnight culture were inoculated into LB to an OD_600_ of approximately 0.09 in 96-well microtiter plates. Plates were incubated with double-orbital shaking at 282 cpm for 24 h at 37 °C in an SynergyH1 or Epoch 2, Biotek, spectrophotometer with OD_600_ measured in each well every 15 min. For the c-M9-CA-te OD600 growth experiments, 1 mL of an LB overnight culture was washed and re-suspended in 1× PBS before inoculation into the c-M9-CA-te to an OD_600_ of approximately 0.08 and growth was monitored as described above. For 90% ascites and 90% serum growth curves bacteria from a fresh overnight culture were diluted in 1× PBS to achieve a starting inoculum of approximately 1 × 10^5^ CFU/mL. Bacteria were enumerated from aliquots removed at designated times by plating serial 10-fold dilutions. A minimum of 3 biological assays with 3 technical repeats were performed and the results were reported as the mean ± the SD.

### Quantitation of capsule

Uronic acid (UA) content was measured as the means to quantitate capsule. The assay was performed largely as described^41^. In brief, 2 mL of LB broth were inoculated for each strain and incubated overnight at 37 °C. The following day, overnight cultures were diluted to an OD_600_ of 0.2, then incubated for 5 h at 37 °C; enumeration of these cultures established that titers were approximately 5 × 10^9^ CFU/mL. Five hundred uL of culture was combined with 100 uL 1% Zwittergent 3–08, heated at 50 °C for 20 min, and centrifuged for 5 min at max speed. Three hundred uL of supernatant was combined with 1.2 mL absolute ethanol and incubated overnight at 4 °C. The next day, the supernatant was decanted following 10-minute centrifugation at 16,000 x g. The pellet was allowed to dry for 10 min, then resuspended in 200 uL diH_2_O, before being combined with 1.2 mL 12.5 mM tetraborate-sulfuric acid solution. After a 10-minute incubation at 100 °C, followed by 10 min on ice, 20 uL of 0.15% 3-phenylphenol was added and incubated at room temp for 5 min. One hundred uL of sample was plated in triplicate and the absorbance measured at 520 nm. Uronic acid concentration was determined via standard curve generated using glucuronolactone. A total of three biological assays with three technical replicates were performed for each strain.

### Phagocytosis assay

J774A.1 macrophages (ATCC TIB-67) were grown in Dulbecco’s modified Eagle’s medium (DMEM) containing L-glutamine, 4.5 g/L glucose, 10% heat-inactivated fetal bovine serum, and Gibco Pen Strep (10,000 units/L penicillin and 10,000 ug/L streptomycin). Upon achieving 90–95% confluency, the cells were washed and harvested by scraping in phosphate buffered saline (PBS). For use in phagocytosis assays cells were resuspended in growth medium lacking penicillin and streptomycin, seeded in 24-well plates at a density of 2.5 × 10^5^ cells/well, and allowed to reattach overnight at 37 °C in 5% CO_2_. Bacterial cultures were grown in 2 mL of LB broth, shaking overnight at 37 °C. The next day, bacterial overnight cultures were diluted to an OD_600_ of 0.05 in fresh LB and grown to mid-logarithmic phase (OD_600_ of approximately 0.4). Mid-logarithmic cultures were then centrifuged at 11,000 x g for 10 min, washed once with PBS, and diluted in DMEM to achieve a concentration of 5 × 10^6^ CFU/mL (Supplementary Fig. 5). Five hundred uL of diluted bacteria were added to each well with a resultant multiplicity of infection of 10 bacteria for every macrophage (calculated based on cell density seeded). The plates were then centrifuged at 170 x g for 5 minutes to increase contact between bacteria and host cells, and incubated for 30 min at 37 °C. Next, wells were washed three times with PBS and incubated with DMEM containing 300 ug/mL gentamicin for 15 min to eliminate extracellular bacteria. Following three washes with PBS, the macrophages were lysed with 0.1% Triton X-100 for 20 min and the lysate underwent serial 10-fold dilutions, which were plated on LB agar to enumerate bacteria. To confirm the bactericidal activity of gentamicin some wells, prior to infection, were incubated with cytochalasin D (2 µg/mL) for 30 min to inhibit bacterial uptake (Supplementary Fig. 5). The number of bacteria phagocytosed was the concentration of surviving bacteria from cytochalasin D-treated wells subtracted from the concentration of surviving bacteria from the corresponding untreated wells.

### CD1 mouse subcutaneous (SQ) challenge infection model/ethics statement

Animal studies were reviewed and approved by the Veterans Administration Institutional Animal Care Committee (IRBNet # 1580121) and the University at Buffalo-SUNY (N/A-Russo1) and were carried out in strict accordance with the recommendations in the guidelines delineated in the “NIH Guide for the Care and Use of Laboratory Animals“(revised 1985) and the “Ethics of Animal Experimentation Statement” (Canadian Council on Animal Care, July 1980) as monitored by the Institutional Animal Care and Use Committee (IACUC). All efforts were made to minimize suffering. Veterinary care for the animals was supplied by the staff of the Veterans Administration Animal Facility under the direction of a fully licensed veterinarian. Studies were performed as described^35^ and reported and performed in accordance with ARRIVE guidelines 2.0. The various challenge inocula and number of outbred CD1 mice (Charles River Laboratories, Wilmington, Massachusetts, United States) used for each strain are delineated in Supplementary Table S4. Animals were closely monitored for 14 days after challenge for the study endpoints, which were survival or severe illness (*in extremis* state/death), which was recorded as a dichotomous variable. Signs that were monitored and which resulted in immediate euthanasia by CO2 narcosis followed by cervical dislocation, methods consistent with the recommendations of the American Veterinary Medical Association Guidelines, included hunched posture, ruffled fur, labored breathing, reluctance to move, photophobia, and dehydration.

### Minimal inhibitory concentration determination (MIC)

Bacterial strains were grown overnight in LB. Bacteria were diluted 1:1,000 in Mueller Hinton II cation adjusted broth (MH) and titers were confirmed by performing serial 10-fold dilutions and enumeration on LB plates. One hundred µL of diluted bacteria were added to a 96-well microtiter plate that contained various concentrations of ceftriaxone in 100 µL of MH with final concentrations ranging from 0.00195 to 32 ug/mL, resulting in a final bacterial concentration of approximately 5 × 10^5^ CFU/mL. Antimicrobial activity was determined by the measurement of optical density (OD_600_). Control wells contained medium only or bacteria without ceftriaxone. Plates were incubated a BioTek spectrophotometer for 20 h, double-orbital shaking (237 cpm (4 mm), slow pace, 37℃) for 20 h at 37 °C. Strains were assessed in parallel to control for inter-test variability. The MIC was defined as the lowest concentration of ceftriaxone that inhibited growth.

### Statistical analyses

*For in vitro* growth curves (Supplementary Figure S3) area under the curve was calculated as described^42^. Desired comparisons between strains for experiments assessing siderophore production (Figs. 2a), mucoviscosity when grown in LB or c-M9-CA-te (Figs. 2b-c and 4b-c), uronic acid quantification (Figs. 3d and 4a), phagocytosis (Figs. 3e and 5a), and in vitro growth curves (Figs. 3a-c and f-g and 5b-c) were made via ordinary one-way ANOVA, using Šidák’s multiple comparisons test (Prism 10.4.2 for MacIntosh, GraphPad Software Inc.).

LD_50_ values were estimated using a logistic regression model as described^43^ Pair-wise comparisons of the dose-response curves were used to generate LD_50_ values. Desired comparisons between LD_50_ values were made by employing a blend of the empirical logit function along with least-squares regression incorporating strain and inoculum factors (CFU/mL) to derive p-values for comparing dose-response curves based on LS-means (Figs. 2d and 5d).

## Supplementary Information

Below is the link to the electronic supplementary material.

Supplementary Material 1

Supplementary Material 2

Supplementary Material 3

Supplementary Material 4

Supplementary Material 5

Supplementary Material 6

Supplementary Material 7

Supplementary Material 8

Supplementary Material 9

Supplementary Material 10

## Data Availability

Both genomic assemblies and raw sequencing data of the isolates analysed in this study are publicly available in NCBI database under the Bio Project number PRJNA1265108 https://www.ncbi.nlm.nih.gov/bioproject/?term=PRJNA1265108.
