# Supplementary figures and images for: Hypervirulent Klebsiella pneumoniae causing aortitis retains its capsule and mucoviscosity and remains genotypically and phenotypically stable over time
Source: Sci Rep. 2025 Nov 13;15:39781. doi: 10.1038/s41598-025-23563-1 (PMC12615786; doi:10.1038/s41598-025-23563-1)

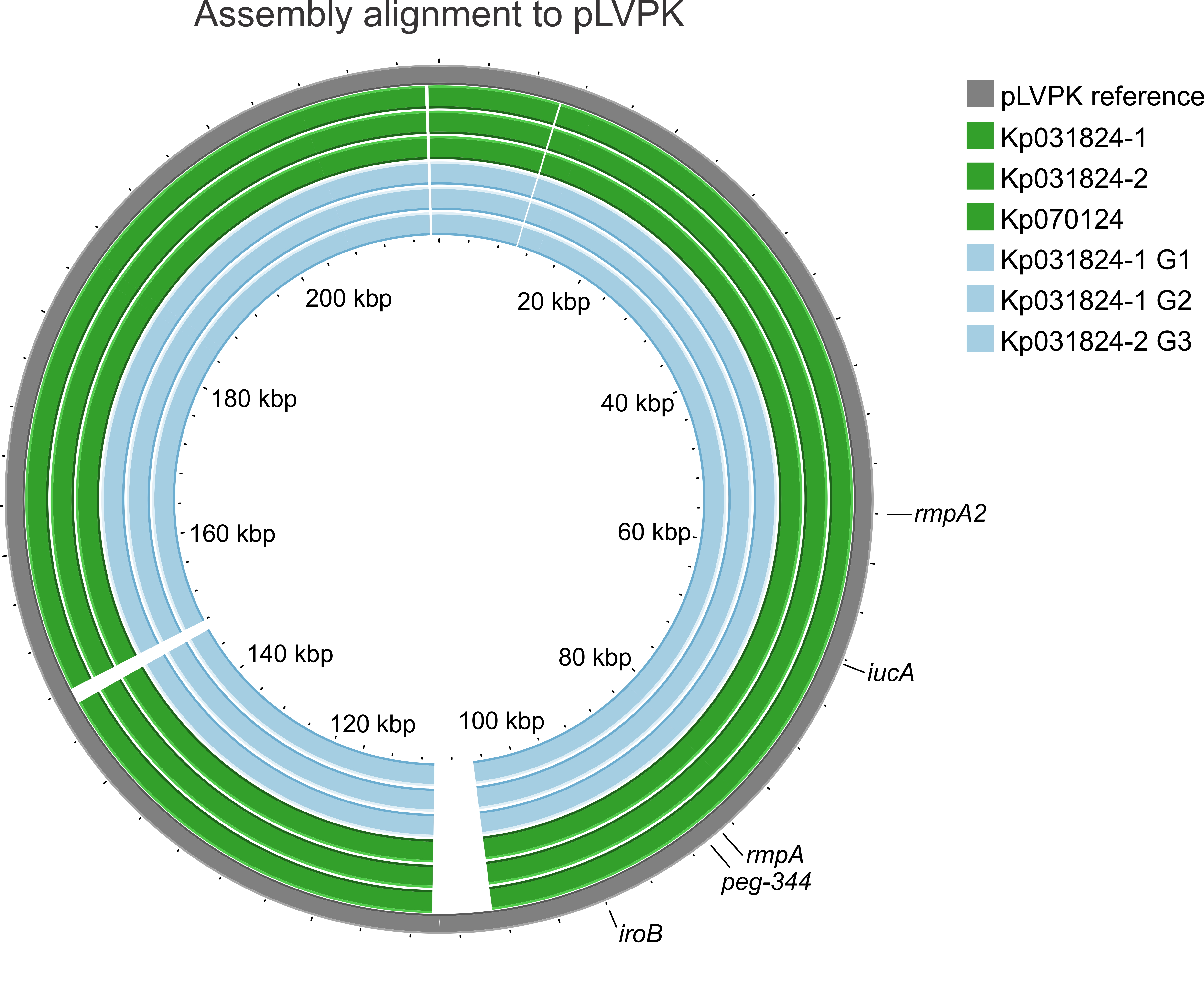

Supplement: Supplementary file 1 — Supplementary Material 1 [file 41598_2025_23563_MOESM1_ESM.jpg]

Threshold of 13

A

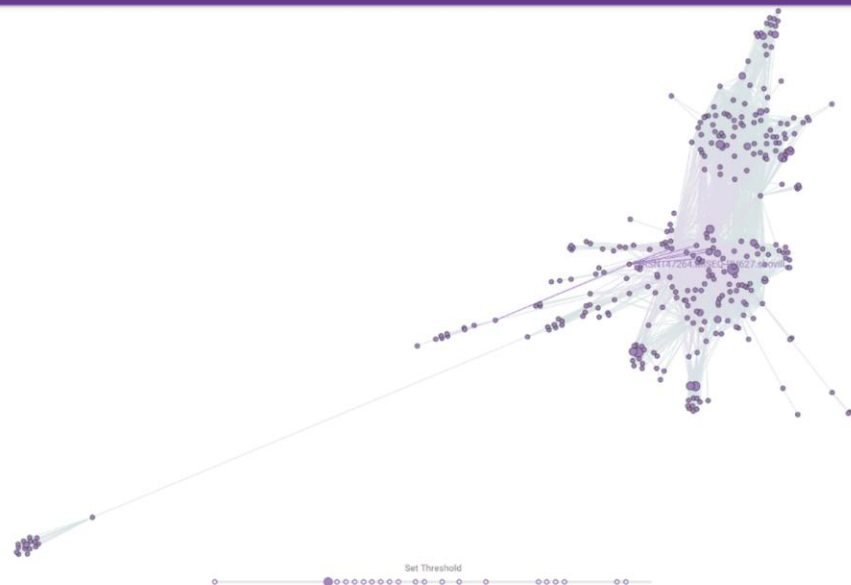

Timeline

C

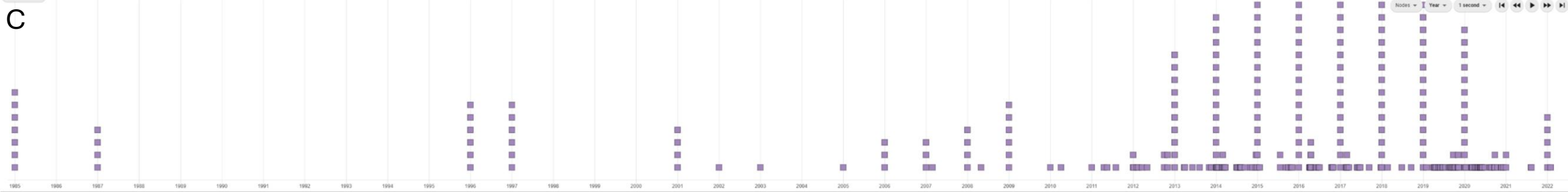

B

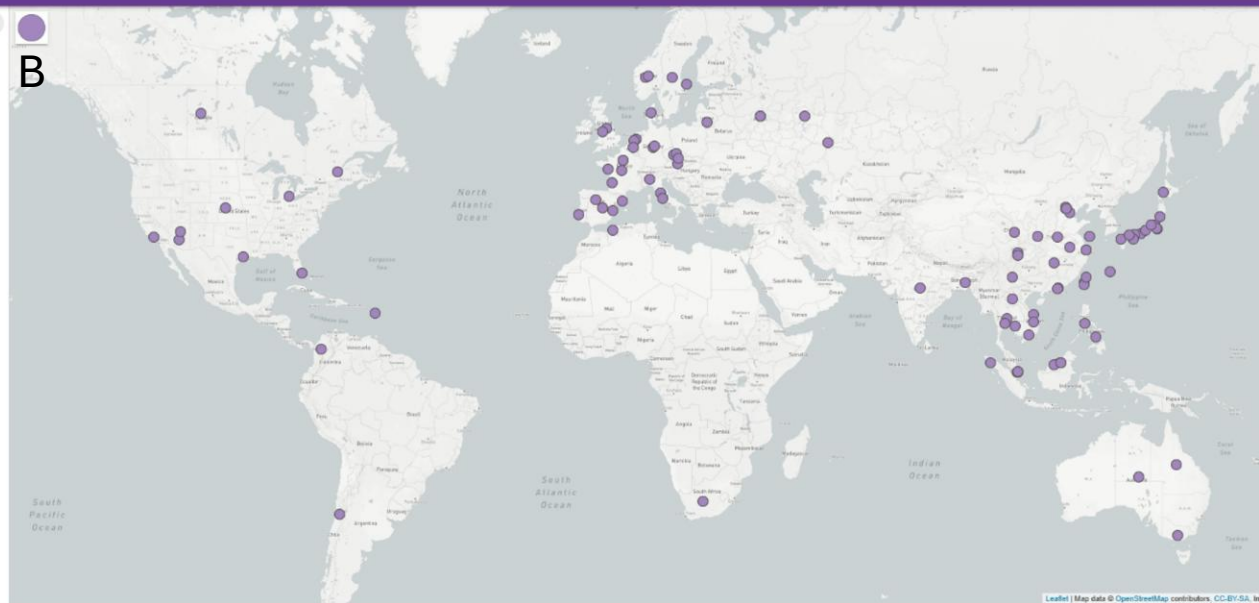

Supplement: Supplementary file 2 — Supplementary Material 2 [file 41598_2025_23563_MOESM2_ESM.pdf]

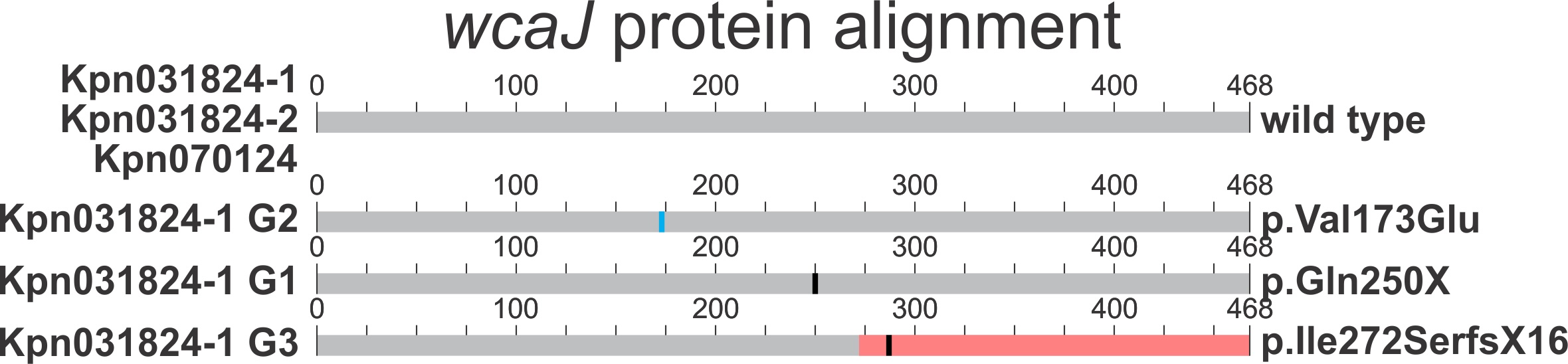

Supplement: Supplementary file 4 — Supplementary Material 4 [file 41598_2025_23563_MOESM4_ESM.jpg]
