# Supplementary material for: Hypervirulent Klebsiella pneumoniae causing aortitis retains its capsule and mucoviscosity and remains genotypically and phenotypically stable over time
Source: Sci Rep. 2025 Nov 13;15:39781. doi: 10.1038/s41598-025-23563-1 (PMC12615786; doi:10.1038/s41598-025-23563-1)
Supplement: Supplementary file 10 — Supplementary Material 10 [file 41598_2025_23563_MOESM10_ESM.pdf]

Supplementary Figure Legends:

Supplementary Figure S1:

Assembly alignment to canonical pLVPK sequence. The three clinical isolates from the patient are represented in green and the three spontaneous capsule minus derivatives are represented in teal. Assemblies of all six isolates possess sequences that align to a significant fraction of pLVPK sequence, including regions that carry the five canonical virulence biomarkers: *iucA*, *iroB, peg-344,* and *rmpA/A2*. Alignment boundaries to the pLVPK sequence indicate that the six isolates likely carry identical or very genetically similar pLVPK-like plasmids.

Supplementary Figure S2:

Genomic relatedness of Kp031824-1 with other ST23 *K. pneumoniae* using the global platform for genomic surveillance PathogenWatch. **(A)** Cluster of genetic relatedness with 352 publicly available isolates (listed in Supplemental table S1), determined at a 13 allelic threshold. (**B**) Map of the geographical origins of 352 isolates clustering Kp031824-1 at the selected threshold. (**C**) Timeline for the date of isolation 352 isolates clustering with Kp031824-1 at the selected threshold.

Supplementary Figure S3:

In vitro growth assessment in LB, che-M9-CA-te, and human serum, ∆56 serum, and ascites. Panels a-b**.** Growth curves for Kp031824-1, Kp031824-2, Kp070124, Kp031824-1 G1, Kp031824-1 G2, and Kp031824-2 G3 in LB and che-M9-CA-te, respectively. Growth was measured by OD_{600} over 24 hours. hvKp1 (hvKp) and MRSN110821 (cKp) were utilized as controls. A minimum of three biological replicates with three technical repeats was performed for each strain. Panels c-e**.** Growth curves for Kp031824-1, Kp031824-2, Kp070124, Kp031824-1

G1, Kp031824-1 G2, and Kp031824-2 G3 in 90% human serum, Δ56 serum, and ascites. Growth was measured via enumeration of colony-forming units over 24 hours. MRSN110821 (cKp) was utilized as a control. A minimum of three biological replicates with two or three technical repeats was performed for all strains in excluding control strains.

Supplementary Figure S4:

Protein alignment of the *wcaJ* gene between the three clinical isolates and each of the three spontaneous capsule minus derivatives. Kpn031824-1 G2 possesses a *wcaJ* variant that differs from wild-type by one amino acid residue at site 173. Kpn031824-1 G1 possesses a *wcaJ* variant with an early stop codon at site 250. KPN031824-1 G3 possesses a *wcaJ* variant with a frameshift mutation beginning at site 272, eventually hitting a stop codon 16 residues downstream of the frameshift. Both stop codon mutations are high impact variants, likely leading to loss of function of *wcaJ*. The missense mutation in Kpn031824-1 G2 has a putative effect on the final protein structure of *wcaJ*, potentially leading to a loss of function or decreased functional efficacy.

Supplementary Figure S5:

Quantification of T_0 bacterial input, T_p bacterial survival, and cytochalasin D (CD) percent survival for phagocytosis assay. Panel a**.** Concentrations of bacteria at T_0 prior to infection of macrophages, measured in CFU/mL via enumeration of colony-forming units on LB agar. An initial target concentration of approximately $5x10^6$ CFU/mL was attained as shown, from which 500 µL of this cell suspension was added to each well, achieving a concentration of $2.5x10^6$ CFU/well, and a multiplicity of infection of 10 bacteria for every macrophage. A minimum of three biological replicates for each strain is shown. Panel b**.** Concentration of surviving bacteria after a 30-minute infection period, 15-minute gentamicin treatment, and 20-minute lysis of

macrophages (time point T_p), via enumeration of colony-forming units on LB agar. Control wells were treated with cytochalasin D (CD) for 30 minutes prior to infection with bacteria. A minimum of three biological replicates with three technical repeats was performed for bacteria that interacted with non-CD treated macrophages. Panel c**.** Survival of bacteria that interacted with CD-treated macrophages as a percentage of surviving bacteria interacted with untreated macrophages. For a given strain, percent survival was calculated by dividing the T_p concentration of bacteria in CD-treated wells by the T_p concentration of bacteria in untreated wells. These data provide insight into CD's effectiveness at inhibiting phagocytosis and the bactericidal activity of gentamicin for a given experiment. A minimum of three biological replicates per condition for each strain is shown. hvKp2 and hvKp2ΔpVir were utilized as controls for phagocytic uptake. All data is presented as the mean ± SD.
